# Utilização de Índices Aterogênicos como Métodos de Avaliação da Doença Aterosclerótica Clínica

**DOI:** 10.36660/abc.20250691

**Published:** 2026-04-17

**Authors:** Muhammed Seyithanoğlu

**Affiliations:** 1 Kahramanmaraş Sütçü İmam University Faculty of Medicine Department of Biochemistry Kahramanmaraş Turquia Kahramanmaraş Sütçü İmam University Faculty of Medicine, Department of Biochemistry, Kahramanmaraş – Turquia

**Keywords:** Aterosclerose, HDL-Colesterol, Características Sexuais, Fatores de Risco, Doenças Cardiovasculares

**Ao Editor,**

A recente publicação de Araujo et al.^1^ fornece informações valiosas sobre a relação entre a aterosclerose e o índice aterogênico do plasma (AIP), o Índice de Risco de Castelli I (CRI-I) e o Índice de Risco de Castelli II (CRI-II). Seus resultados, que mostram valores elevados dos índices em pacientes com doença aterosclerótica em comparação com os controles, ressaltam o papel potencial dessas relações derivadas de lipídios como preditores práticos e de baixo custo do risco cardiovascular em uma população brasileira.

No entanto, após análise detalhada, uma característica metodológica parece merecer maior atenção. O grupo com aterosclerose em sua coorte apresentou uma proporção notavelmente menor de participantes do sexo feminino em comparação com o grupo controle (47,1% versus 62,8%). Dado que esses índices são derivados de frações lipídicas amplamente determinadas pelas concentrações de colesterol HDL (HDL-C) e que o HDL-C é profundamente influenciado pelo sexo, esse desequilíbrio demográfico pode potencialmente modular os resultados relatados. A diferença observada entre os grupos, portanto, pode não refletir exclusivamente a carga aterosclerótica; também pode representar o impacto de variações biológicas relacionadas ao sexo.

É um fato bem estabelecido em laboratório que as concentrações de HDL-C tendem a ser mais altas em mulheres do que em homens, conforme descrito em textos de referência bioquímica padrão.^2^ As diferenças nos perfis de hormônios sexuais, distribuição de gordura corporal e metabolismo lipídico hepático contribuem para essa variação. Consequentemente, a composição desigual de sexos entre os grupos de um estudo pode afetar inadvertidamente os cálculos baseados em HDL-C e, por extensão, quaisquer índices que dependam de HDL-C como denominador. Isso introduz uma importante fonte de confusão que justifica ajuste ou estratificação explícitos nas análises estatísticas.

Em nossa própria investigação envolvendo 419 indivíduos submetidos à angiotomografia de artérias coronárias,^3^ avaliamos diversos marcadores lipídicos não tradicionais — a saber, a relação monócito-HDL (MHR, do inglês, *monocyte-to-HDL ratio*), AIP, CRI-I e CRI-II — para explorar sua associação com a doença arterial coronariana (DAC). Para garantir uma análise equilibrada e abrangente, utilizamos dois esquemas de classificação complementares:

1. um sistema CAD-RADS de seis níveis que permitiu uma avaliação detalhada das tendências ao longo da progressão da DAC; e
2. um modelo binário (CAD-RADS 0 a 2 versus 3 a 5) concebido para avaliar a potencial necessidade de angiografia coronária invasiva.

Nas seis categorias de CAD-RADS, não foi detectada nenhuma diferença significativa nos valores de MHR, AIP, CRI-I ou CRI-II. Porém, dentro do modelo binário, esses índices pareceram mais elevados entre os pacientes do grupo com CAD-RADS 3 a 5 (p = 0,008 a 0,029). Notavelmente, a distribuição por sexo também diferiu significativamente entre esses dois subgrupos e, quando foram realizadas análises específicas por sexo, a significância estatística dos índices desapareceu, indicando que o desequilíbrio entre os sexos teve um efeito mensurável nos resultados.

Em conjunto, nossas observações indicam que a aparente utilidade preditiva das relações lipídicas relacionadas ao HDL-C — tais como AIP, CRI-I, CRI-II ou MHR — pode ser influenciada pela distribuição por sexo, especialmente em coortes com predominância masculina. Agradecemos a contribuição de Araújo et al.^1^ e concordamos que esses índices não tradicionais são promissores como marcadores cardiovasculares acessíveis. No entanto, recomendamos que pesquisas futuras controlem explicitamente as diferenças de HDL-C relacionadas ao sexo para determinar com mais precisão o valor preditivo independente desses índices na aterosclerose.
