# Supplementary material for: Utilização de Índices Aterogênicos como Métodos de Avaliação da Doença Aterosclerótica Clínica
Source: Arq Bras Cardiol. 2026 Apr 17;123(3):e20250691. [Article in Portuguese] doi: 10.36660/abc.20250691 (PMC13128218; doi:10.36660/abc.20250691)
Supplement: Translation

# Translation: Use of Atherogenic Indices as Assessment Methods of Clinical Atherosclerotic Diseases

**DOI:** 10.36660/abc.20250691i

**Authors:** Muhammed Seyithanoğlu

**Affiliations:** 1 Kahramanmaraş Türkiye Kahramanmaraş Sütçü İmam University Faculty of Medicine, Department of Biochemistry, Kahramanmaraş – Türkiye

**Keywords:** Atherosclerosis, HDL Cholesterol, Sex Characteristics, Risk Factors, Cardiovascular Diseases

**To the Editor,**

The recent publication by Araujo et al.^1^ provides valuable insight into the relationship between atherosclerosis and the atherogenic index of plasma (AIP), Castelli Risk Index I (CRI-I), and Castelli Risk Index II (CRI-II). Their results, showing elevated index values in patients with atherosclerotic disease compared with controls, underscore the potential role of these lipid-derived ratios as practical and inexpensive predictors of cardiovascular risk within a Brazilian population.

Nevertheless, upon detailed examination, one methodological feature appears to merit further consideration. The atherosclerosis group in their cohort contained a noticeably smaller proportion of female participants compared to the control group (47.1% versus 62.8%). Given that these indices are derived from lipid fractions largely determined by HDL cholesterol (HDL-C) concentrations and that HDL-C is profoundly influenced by sex, such demographic imbalance could potentially modulate the reported results. The observed difference between groups, therefore, may not exclusively reflect atherosclerotic burden but could also represent the impact of sex-related biological variation.

It is a well-established laboratory fact that HDL-C concentrations tend to be higher in women than in men, as described in standard biochemical reference texts.^2^ Differences in sex hormone profiles, body fat distribution, and hepatic lipid metabolism all contribute to this variation. Consequently, unequal sex composition across study arms can inadvertently affect HDL-C–based calculations and, by extension, any indices that rely on HDL-C as a denominator. This introduces an important source of confounding that warrants explicit adjustment or stratification in statistical analyses.

In our own investigation involving 419 individuals undergoing coronary computed tomography angiography,^3^ we assessed several non-traditional lipid markers—specifically, the monocyte-to-HDL ratio (MHR), AIP, CRI-I, and CRI-II—to explore their relationship with coronary artery disease (CAD). To ensure a balanced and comprehensive analysis, we utilized two complementary classification schemes:

1. a six-level CAD-RADS system that enabled a detailed evaluation of trends across progressive CAD severity; and
2. a binary model (CAD-RADS 0 to 2 versus 3 to 5) designed to assess the potential necessity of invasive coronary angiography.

Across the six CAD-RADS categories, no significant difference was detected for MHR, AIP, CRI-I, or CRI-II values. However, within the binary model, these indices appeared higher among patients in the CAD-RADS 3 to 5 group (p = 0.008 to 0.029). Notably, sex distribution also differed significantly between these two subgroups, and when sex-specific analyses were performed, the statistical significance of the indices disappeared, indicating that sex imbalance had a measurable effect on the results.

Collectively, our observations indicate that the apparent predictive utility of HDL-C–related lipid ratios—such as AIP, CRI-I, CRI-II, or MHR—may be influenced by sex distribution, especially in cohorts with male predominance. We appreciate the contribution of Araujo et al.^1^ and agree that these non-traditional indices hold promise as accessible cardiovascular markers. Nevertheless, we recommend that future research explicitly control for sex-related HDL-C differences to more accurately determine the independent predictive value of such indices in atherosclerosis.
